# Modeling Topics in DFA-Based Lemmatized Gujarati Text

**DOI:** 10.3390/s23052708

**Published:** 2023-03-01

**Authors:** Uttam Chauhan, Shrusti Shah, Dharati Shiroya, Dipti Solanki, Zeel Patel, Jitendra Bhatia, Sudeep Tanwar, Ravi Sharma, Verdes Marina, Maria Simona Raboaca

**Affiliations:** 1 Department of Computer Engineering, Vishwakarma Government Engineering College, Chandkheda, Ahmedabad 382424, India; 2 Department of Computer Science and Engineering, Institute of Technology, Nirma University, Ahmedabad 382481, India; 3 Ravi Sharma, Centre for Inter-Disciplinary Research and Innovation, University of Petroleum and Energy Studies, Dehradun 248001, India; 4 Faculty of Civil Engineering and Building Services, Department of Building Services, Technical University of Gheorghe Asachi, 700050 Iasi, Romania; 5 Doctoral School, University Politehnica of Bucharest, Splaiul Independentei Street No. 313, 060042 Bucharest, Romania; 6 National Research and Development Institute for Cryogenic and Isotopic Technologies—ICSI Rm. Vâlcea, Uzinei Street, No. 4, P.O. Box 7 Râureni, 240050 Râmnicu Vâlcea, Romania

**Keywords:** topic models, Gujarati text lemmatization, Latent Dirichlet Allocation, poor quality topics, overly general topics

## Abstract

Topic modeling is a machine learning algorithm based on statistics that follows unsupervised machine learning techniques for mapping a high-dimensional corpus to a low-dimensional topical subspace, but it could be better. A topic model’s topic is expected to be interpretable as a concept, i.e., correspond to human understanding of a topic occurring in texts. While discovering corpus themes, inference constantly uses vocabulary that impacts topic quality due to its size. Inflectional forms are in the corpus. Since words frequently appear in the same sentence and are likely to have a latent topic, practically all topic models rely on co-occurrence signals between various terms in the corpus. The topics get weaker because of the abundance of distinct tokens in languages with extensive inflectional morphology. Lemmatization is often used to preempt this problem. Gujarati is one of the morphologically rich languages, as a word may have several inflectional forms. This paper proposes a deterministic finite automaton (DFA) based lemmatization technique for the Gujarati language to transform lemmas into their root words. The set of topics is then inferred from this lemmatized corpus of Gujarati text. We employ statistical divergence measurements to identify semantically less coherent (overly general) topics. The result shows that the lemmatized Gujarati corpus learns more interpretable and meaningful subjects than unlemmatized text. Finally, results show that lemmatization curtails the size of vocabulary decreases by 16% and the semantic coherence for all three measurements—Log Conditional Probability, Pointwise Mutual Information, and Normalized Pointwise Mutual Information—from −9.39 to −7.49, −6.79 to −5.18, and −0.23 to −0.17, respectively.

## 1. Introduction

Topic modeling is statistical modeling for uncovering abstract “topics” hidden in a massive text collection. For example, Latent Dirichlet Allocation (LDA) infers the topics in a text collection [1]. Linguistic field researchers have shown great interest in techniques for discovering a smaller set of word clusters (known as topics) that represents the whole corpus without losing its significance. The set of techniques for modeling topics in domains are Latent Semantic Analysis (LSA) [2], probabilistic Latent Semantic Analysis (pLSA) [3], followed by LDA.

Practitioners have been using topic models to explore the semantic and statistical properties of text corpora. They have successfully applied the technique to a variety of text domains such as scientific and research article corpora [4,5,6,7], health and clinical areas [8,9,10,11], software domains [12,13,14,15,16,17,18,19], etc. The application of topic models has also been expanded to non-textual data such as (1) a video corpus for person re-identification [20] and human-action recognition [21], (2) an image collection to reorganize images into groups based on their quality [22], and (3) an audio dataset for retrieving audio using the features of audio [23]. Additionally, in various research tasks, a hierarchy of topics has been modeled as opposed to flattened topic modeling [24,25,26,27,28,29]. Additionally, topic modelers have also explored short texts, such as tweets on Twitter [30,31,32,33,34,35,36] or customer reviews [37,38,39], for discovering hidden thematic structures. The topic model presupposes that each document in the collection is a combination of various topics and that each is a combination of words. A probability distribution across the vocabulary words is subjective. Table 1 illustrates themes derived from a collection of English newspapers.

One of the topic models’ byproducts—topics—can be used either directly in information extraction or as an intermediate output that serves as an input for the subsequent task phase.

Despite numerous extensions worldwide, some areas of LDA still call for more reflection. Preprocessing techniques like stopword removal, stemming, and lemmatization must be created for many languages. Although they can seem like a straightforward component of text summarization, their existence or absence has a significant impact on the output since a thorough evaluation of these preprocessing activities results in more meaningful topics in a shorter period of time. However, the enormous breadth of the vocabulary may come from excluding such stages. The inference procedure consequently requires greater processing resources. Furthermore, less emphasis has been placed on linguistic features like synonyms, polysemy, homonymy, hyponymy, and so forth. These language traits improve the issues’ semantic coherence. Also crucial to topic modeling are the preprocessing elements. Language-specific preprocessing is frequently used in NLP research assignments. Instead of getting rid of language-specific stopwords, Schofield et al. suggested topic models preceded by corpus-specific stopwords [40].

### 1.1. Motivation

Stochastic topic models uncover the latent topical structure, which is smaller in size and easier to understand. However, they need to improve the output at times. The vocabulary size in the text collection of a morphologically rich language increases with the increase in the size of the corpus. It is a fact that topic models transform a mammoth text collection into a manageable topical subspace that is easier to interpret; however, the training phase of LDA may prove itself computationally expensive in the case of the huge size of the vocabulary. This phenomenon is because the statistical inference process continuously refers to the vocabulary. If we reduce the vocabulary size without disturbing the quality of the corpus, the inference process computation cost can be decreased. Moreover, the semantic coherence of topics could also be increased remarkably.

### 1.2. Contribution of the Paper

The main lines of the contribution process of this paper consist of the following:

- We propose a DFA-based lemmatization approach for Gujarati text.
- We show that lemmatization of Gujarati text reduces lemmas to their base words to curtail the vocabulary size notably.
- The topic can be inferred quicker in a lemmatized Gujarati corpus, resulting in improvement in the interpretability of the discovered topics at the same time.
- The semantic coherence measurement has been performed by three methods to analyze it precisely.
- Additionally, we have used two different measurement methods to show the distance between topics. We proved that meaningful and precise topics fall far from overly general topics. The distance of the meaningful topics from the token distribution of the entire corpus is also larger compared to that for overly general topics.

### 1.3. Organization of the Paper

The rest of the paper has been organized as follows: Section 2 covers the literature study relevant to the proposed methodology. Then, Section 3 explains the DFA-based approach for lemmatization. Following this, Section 4 briefs about topic inference techniques with their parameters. It depicts some relevant figures of automata. Additionally, a few rules for the first word of the sentence and the rest of the sentences have been shown in tabular format. Next, Section 5 discusses the experimental setup and measurement techniques. Finally, Section 6 displays the experimental findings and their comparison.

### 1.4. Scope of the Paper

The paper explains the effect of lemmatized text for modeling topics. The technique applies to Gujarati text specifically and to the dataset under study; changes may be needed for it to work more efficiently on another dataset of Gujarati text.

## 2. Related Work

Although preprocessing of the corpus has been considered a very obvious phase, it exhibits challenges when dealing with languages that have a rich morphology. This is because the stemming and lemmatization process differs from one language to another. Most of the topic modeling research tasks target the corpus of English text, as the resources are available for preprocessing. There are several earlier works about learning topic models preceded by stemming or lemmatization. Brahmi et al. modeled the topics in stemmed Arabic text [41]. They achieved two main objectives: first, to extract the stem from the morphemes, and second, to infer topics from Arabic text using LDA. Lu et al. investigated the impact of removing frequent terms from the vocabulary [42]. They measured the computational overhead for different numbers of topics. Designing lemmatization for many languages has captured the attention of linguists. For languages like Hindi [43], academics have created lemmatization of the Indian language, such as Bengali [44] and Tamil [45]. Likewise, Al-Shammari et al. proposed Arabic lemmatization techniques [46] and stemming in another work [47]. Roth et al. also designed an Arabic lemmatizer with feature ranking [48]. The European languages lemmatization approaches include French [49], Polish [50], Finnish [51], Czech [52], German, Latin [53], and Greek [54]. Similarly, the Kazakh language [55], Turkish [56], and Urdu [57,58] have also been considered for lemmatization. Table 2 shows the lemmatization work for the different languages.

## 3. Deterministic Finite Automata (DFA) Based Gujarati Lemmatizer

**Figure 1:**
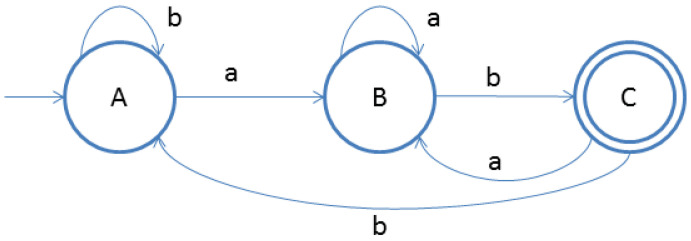
A DFA accepting the strings ends with ‘ab’.

DFAs, also known as deterministic finite automata, are finite state machines that accept or reject character strings by parsing them through a sequence specific to each string. It is said to be “deterministic” when each string, and thus each state sequence, is distinct. Each input symbol in a DFA moves to the next state that can be predicted as a string of symbols is parsed via DFA. For example, if you want to parse all strings in the alphabet a,b that end with ‘ab,’ then Figure 1 depicts the DFA that accepts only the correct strings.

There are two approaches for generating a root word from its inflected word: stemming and lemmatization. Stemming is the method to remove the suffixes/prefixes of the words to get the root words [61]. Lemmatization refers to deriving the root words from the inflected words. A lemma is the dictionary form of the word(s) in the field of morphology or lexicography. To achieve the lemmatized forms of words, one must analyze them morphologically and have the dictionary check for the correct lemma. As a result, the lemmatized word always conveys a proper meaning, while a stemmed word may come out without any meaning. Table 3 explains the difference between stemming and lemmatization. It can be observed that a stemmed word may or may not be the dictionary word, while a lemma must be a dictionary word.

**Figure 2:**
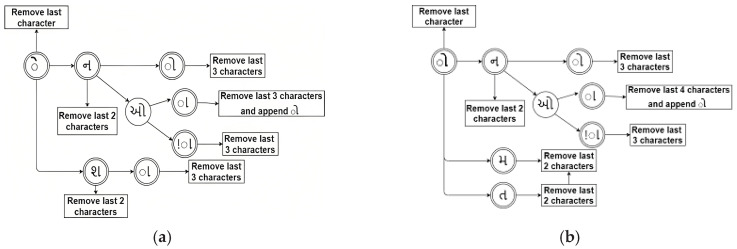
Deterministic finite automata for lemmatization for rules 1 and 2. (**a**) Rule 1; (**b**) Rule 2.

**Figure 3:**
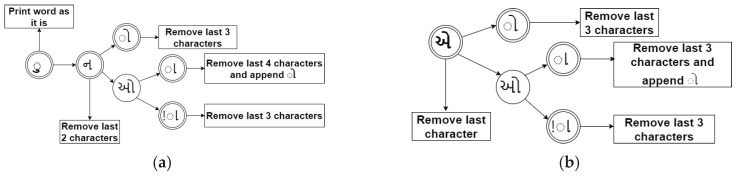
(**a**) Rule 3; (**b**) Rule 4. Deterministic finite automata for lemmatization for rules 3 and 4.

For example, using the continuous bag-of-words model, word embedding applications consider the N (size of windows) surrounding context words to predict the word. Hence, before vocabulary building takes place, the words of the text collection must be preprocessed in terms of stopwords removal, stemming, lemmatizing, etc. This results in the shrinkage of vocabulary size and speeds up the model-building process. Similarly, morphologically, topic modeling in the text of the rich language needs to process a massive vocabulary during the topic formation process. One must apply the lemmatization technique to the corpus to have a reduced vocabulary size. This paper discusses this issue, considering Gujarati (the 26th most widely spoken language in India by the number of native speakers [62]) for examination. Table 4 and Table 5 enlist rules for the lemmatization of inflectional forms. Besides, Figure 2a,b depict rule 1 and rule 2 of Table 4 respectively. Similarly, Figure 3a,b depict rule 3 and rule 4 of Table 4 respectively.

In previous work for normalizing the word forms in Gujarati, the stemming approach has received attention from linguistic researchers. Patel et al.  prepared a suffix list and incorporated it into the stemming process. They targeted to get rid of only suffixes of the inflectional words. Likewise, Suba et al. proposed an extended version of stemmer, which is lightweight for suffix removal and a heavyweight rule-base stemmer [63]. Ameta et al. also suggested a similar kind of lightweight stemmer [64]. Aswani et al. offered a morphological study for inflectional forms of the Hindi language, which was extended to the Gujarati language [65]. In all previous approaches, authors have focused on suffix removal to reduce the word to its root. We perform mainly three operations for transforming inflectional forms to a lemma: removing suffixes, appending suffixes, and removal followed by appending of characters.

For the Gujarati language, lemmatization is more accurate in transforming the inflected word into the root word. It involves not only the removal of suffixes but also appending some pattern or characters to the inflected word or the operations one after another. There is a remarkable set of research tasks stemming from the Gujarati language, such as hybrid stemmer of Gujarati [66], Gujarati lightweight stemmer [64], and rule-based stemmer [63]. However, research on lemmatization has not gained much consideration comparatively.

Apart from the research mentioned above, there is hardly any work found in the literature that directly addresses the problems of lemmatization in the Gujarati language. In this article, we propose an automata-based approach for lemmatizing Gujarati text. Besides the automata-based approach, list-based (known as rule-based) and hash-based approaches have also been explored for lemmatization across various languages. We aim to design an automata-based lemmatizer that can transform different inflectional forms to their root word with less computational complexity than the list-based approach.

The different inflectional words are generated by applying one or more transformation rules to their respective dictionary form or lemma. An inflectional word can be formed by appending a valid suffix to its lemma (’મહાપુરુષ’ can form the lemma ’મહાપુરુષોથી’ by appending the suffix ’ઓથી’). Moreover, sometimes removal followed by addition may result in the formation of a valid inflectional form (’છોકરો’ can form the lemma’છોકરાઓનુ’) by appending the suffix ’નુ’) or sometimes none of the mentioned cases. Lemmas can be derived by reversing the corresponding process of generation of inflectional forms from their root words.

## 4. Latent Dirichlet Allocation (LDA)

In Latent Dirichlet Allocation, each document in the corpus of M document is modeled as a multinomial distribution of K hidden topics, and each topic is a multinomial distribution of the vocabulary of V words. Topic modeler inputs the number of topics, K. The document-topic distribution $\theta_{d}$ is drawn from Dirichlet distribution Dir [α], where α is a hyperparameter vector variable with the value $\left(\alpha_{1},\alpha_{2},\cdots ,\alpha_{k}\right)$, which can be estimated. In the same way, the topic–word distribution, $\phi_{k}$, is drawn from the Dirichlet distribution Dir [β].

**Figure 4:**
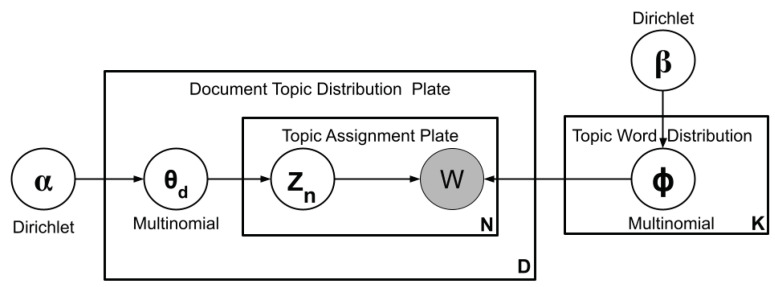
Plate notation for LDA generative algorithm [1].

The Latent Dirichlet Allocation can be represented graphically by the Bayesian network. The plate notation of LDA has been depicted in Figure 4. The node represents the random variable, and the edge represents the influence of one variable on another. The complete document generative process $\theta_{d}$ and $\phi_{k}$ has been shown in Algorithm 1. For the $n_{th}$ word of document d, a topic assignment $Z_{n,d}$ is drawn from $\theta_{d}$, and a word identity is drawn from the corresponding topic, $\phi_{W|Z_{d}}$. Henceforth, the whole generative process is given by

$$\begin{array}{c} \theta_{d}∼Dir\left(\alpha \right)\;Z_{d,n}∼Mul\left(\theta_{d}\right)\\ \phi_{k}∼Dir\left(\beta \right)\;W_{d,n}∼Mul\left(\phi_{k}\right) \end{array}$$

**Algorithm 1:** Generative algorithm for LDA. 
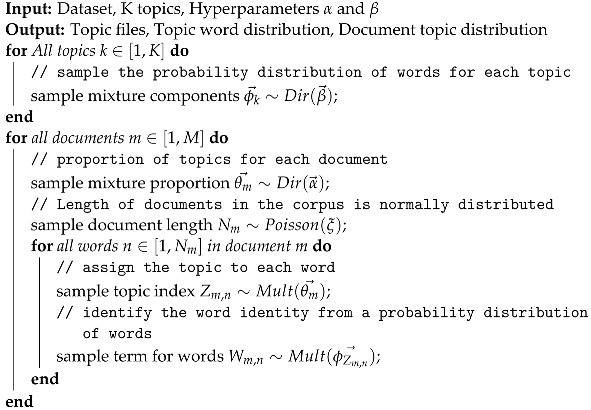


## 5. Experimental Setup

We used the TDIL dataset to assess our lemmatization approach’s efficiency. The dataset was made available with Part-of-Speech (PoS) tagging, but we wanted to consider something other than PoS tagging, as it is optional. Therefore, we filtered the PoS tags to get a compatible dataset for our experimental purposes. We experimented with evaluating two metrics: the improvement in the semantic coherence of topics and the success rate of lemmatization. Initially, the lemmatization process was evaluated in terms of accuracy. We provided the dataset and measured how many words were correctly lemmatized and how many were lemmatized incorrectly. We also considered words that did not need any lemmatization separately. In other words, no rule should apply to such words.

### 5.1. Preprocessing and Vocabulary Size: An Analysis

Although preprocessing of the dataset is a self-explanatory part of the text analysis, it may uncover the detailed statistical properties of the corpus under study. Moreover, preprocessing is also language-dependent. Hence, a specific set of preprocessing steps is required for a given language to know the values of some critical parameters such as the number of tokens in the corpus, vocabulary size, type-to-token ratio (TTR), etc. In TTR, tokens denote the total number of words in a corpus regardless of how often they are repeated. The term “type” concerns the number of distinct words in a corpus. We performed preprocessing for the dataset under study. The preprocessor component comprises several steps, as depicted in Table 6. As shown in Table 6, stopwords removal remarkably reduced the size of the dataset in terms of the total number of tokens and discrete words. We compiled a list of more than 800 Gujarati language stopwords to eliminate them.

The punctuation marks and alphanumeric words are typical cases in many languages. However, Gujarati text might contain Gujarati and English digits blended in the text, so we have taken care to remove the digits of both languages. We also found several words in alphanumeric form, so we transformed them into alphabetic forms by removing mixed digits from such words. However, they are rare in number, and this did not decrease the number of tokens and vocabulary size. The next step is particular to Gujarati text, as the Gujarati corpus contains single-letter words. Table 7 depicts the most frequent single-letter words in the Gujarati language. These words do not contribute to topic modeling and are not part of the stopwords. The crucial part is that we performed lemmatization on the resultant corpus. It reduced the inflectional forms to their root words, known as the lemma.

**Figure 5:**
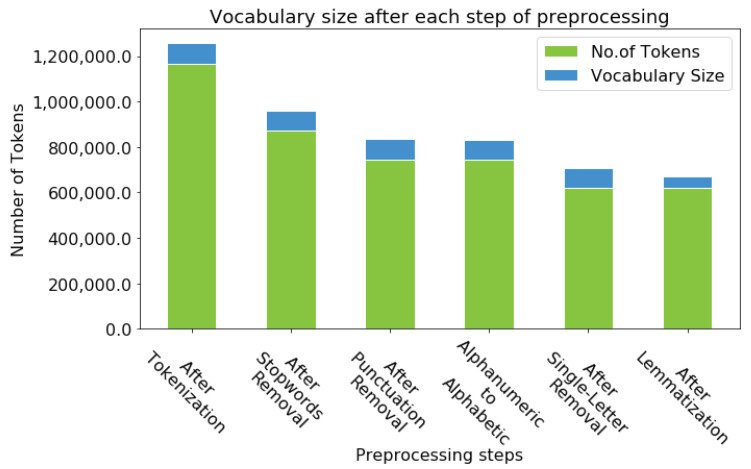
Size of vocabulary.

We have achieved a remarkable reduction in the vocabulary size, as shown in Figure 5. Moreover, after each preprocessing step, one can observe a notable decrease in the number of tokens. Most importantly, the lemmatization left the vocabulary size at 8.07% of the total number of tokens. The vocabulary size was 7.29% before any preprocessing action, but the number of tokens columns was very high. That itself could lead to the heavy computation of inference of the topic. Meanwhile, there is a negligible reduction in vocabulary after the removal of digits and the transformation of alphanumeric words to alphabetic.

As shown in Figure 5, although the lemmatization process does not reduce the number of tokens, it reduces vocabulary size by 58% because instead of eliminating the tokens from the collection, it transforms them to their respective lemma form. Similarly, there were several alphanumeric words in the corpus. We removed the blended digits from those alphanumeric words, leaving behind the alphabetic words. The alphanumeric words might occur due to typing errors. However, we did not perform any tasks for removing the words that occur less frequently than some number N; for example, when N is 3. Other authors have removed words with some lower and upper bounds in frequency in most information retrieval research. For example, the words that occur fewer than three times and more than 500 times are to be removed. In most cases, these words get removed in one of the previous preprocessing steps, such as stopword elimination or single-letter word removal.

### 5.2. Evaluation of the Proposed Lemmatizer Approach

To evaluate the technique, we prepared a lemma-annotated dataset constructed from the dataset itself. This lemma-annotated dataset was constructed with the help of words found frequently in articles based on terms that appear in all four categories of the dataset. They are dictionary words. As mentioned earlier, we did not include single-letter words, stopwords, or punctuation marks in the lemma-annotated dataset, as they have been removed from the preprocessed dataset. We took 2000 lemmas for the experiment, achieved by stratified sampling. On applying the lemmatizer, we achieved 78.6% accuracy. The outcome of the experiment is regardless of Part-of-Speech tagging.

### 5.3. Overly General Topics

Several decision criteria could identify overly general topics. A topic model may consist of different categories of overly general topics. An extensive topic needs to make sense thematically. These themes include a collection of words that have no semantic connection to one another, to put it simply. For example, a topic might cover a significant fraction of the words in the vocabulary. Table 8 depicts an overly general topic, which comprises 11% of vocabulary words. Such topics are very general and do not convey any specific concept. On the other hand, an interpretable topic comprises semantically relevant words. Therefore, one can find some meaning in it, as shown in Table 9. A meaningful topic contains a tiny fraction of the words in the vocabulary, such as 1% to 2%; on the other hand, a few topics may be very common, as they are present in many documents. Furthermore, uninterpretable topics can also be identified by the number of tokens assigned to the top-N words of the topic. Therefore, the word length, i.e., the average number of characters present in the words of the topic, plays a significant part in determining the interpretability of the topic.

#### Distance from a Global Corpus-Level Topic

A topic is a probability distribution of words in the vocabulary of a corpus. Each word in the corpus follows with a specific probability. When topics are inferred from the corpus, the words in the topic also carry the probability values. Here topics are soft clusters so that a word may appear in more than one topic with different probability values. Ideally, words forming the topic are semantically relevant to one other.

### 5.4. Semantic Coherence Measurement Methods

#### 5.4.1. Pointwise Mutual Information (PMI)

To calculate the collocation, PMI might be used. First, though, it serves as a statistical indicator of the proximity of two words. To track the co-occurrence, we changed the sliding window’s word count to 5. The PMI of each set of provided word pairs is then determined by computing (word${}_{1}$, word${}_{2}$). The PMI of any two terms in a topic model has calculated the difference between the likelihood of their co-occurrence given their joint probability distribution and their discrete distributions, assuming that events are unrelated to one another [67,68,69]. It can be written mathematically, as shown.

(1) $$\mathrm{PMI}\left({\mathrm{word}}_{1}\;,{\mathrm{word}}_{2}\right)=\log\frac{P({\mathrm{word}}_{1},{\mathrm{word}}_{2})}{P\left({\mathrm{word}}_{1}\right)P\left({\mathrm{word}}_{2}\right)}$$

The word order does not affect the PMI score for that pair of words. The measurements for PMI (word${}_{1}$, word${}_{2}$) and PMI remain symmetric (word${}_{2}$, word${}_{1}$). The explanation is that since documents are viewed as a “bag of words” (BOW), the sequence in which words appear does not matter. The word order should be emphasized in the reference corpus too. Both positive and negative values could be assigned to the PMI score. If the PMI value is zero, the words are considered to have no relationship with the reference corpus. On the other hand, PMI is greatest when there is a close relationship between the terms.

#### 5.4.2. Normalized Pointwise Mutual Information (NPMI)

The extension of the PMI method is Normalized PMI. It is similar to PMI except that the score of NPMI takes a value between [−1,+1], in which –1 conveys no occurrence together, 0 indicates independence, and 1 indicates complete co-occurrence [68,69].

(2) $$\mathrm{NPMI}\left({\mathrm{word}}_{1},\;{\mathrm{word}}_{2}\right)=\frac{\mathrm{PMI}({\mathrm{word}}_{1},\;{\mathrm{word}}_{2})}{-\log P({\mathrm{word}}_{1},\;{\mathrm{word}}_{2})}$$

#### 5.4.3. Log Conditional Probability (LCP)

Log conditional probability (LCP) is one-sided, while PMI is a symmetric coherence measurement.

(3) $$\mathrm{LCP}\left({\mathrm{word}}_{1},\;{\mathrm{word}}_{2}\right)=\log\frac{P({\mathrm{word}}_{1},\;{\mathrm{word}}_{2})}{P({\mathrm{word}}_{2})}$$

### 5.5. Distance Measurement Methods

The divergence of the topics from some predefined, overly general topic types has been measured. There are two types of metrics for cluster analysis. Supervised evaluation metrics use the labeled samples. On the other hand, unsupervised evaluation does not check the accuracy of the learned model. In this paper, several divergence measures are used to check the efficacy of the proposed techniques.

#### 5.5.1. Hellinger Distance

Hellinger distance between two discrete probability distributions P and Q can be defined as,

(4) $$\mathrm{HD}\left(P,Q\right)=\frac{1}{\sqrt{2}}\sqrt{\sum_{i=1}^{k}{\left(\sqrt{p_{i}}-\sqrt{q_{i}}\right)}^{2}}$$

where $P=\left(p_{1},p_{2},\cdots p_{k}\right)$ and $Q=\left(q_{1},q_{2},\cdots q_{k}\right)$.

#### 5.5.2. Jaccard Distance

The Jaccard similarity measures the similarity between finite sample sets. It is the intersection of sets divided by the union of sample sets. Here, cardinality represents the number of elements in a set, denoted by |a|. Suppose you want to find Jaccard’s similarity between two sets, a and b. It is the ratio of the cardinality of a ∪ b and a ∩ b.

(5) $$\begin{array}{c} J(a,\;b)=|\mathrm{Intersection}(a,\;b)|/|\mathrm{Union}(a,b)| \end{array}$$

Although it seems very simple, it applies to the topic modeling. First, it fetches out the standard terms between two topics and the entire distinct terms. Then, it takes the ratio of common and distinct terms; the results serve as the Jaccard similarity. Finally, by taking the complement, likewise in cosine, the Jaccard distance can be measured.

## 6. Results

### 6.1. Distance Measurement from Global Topic

The distance measurement mentioned above is used to analyze the quality of topic models. We framed the global topic as the frequency distribution of words in the vocabulary. We used the Hellinger distance to compute the distance of topics from the global topic. The distance between the topic model and the topic model with lemmatized terms has been compared. An experiment examined the effect of lemmatization on vocabulary size and inferred topic quality. We inferred 100 topics by iterating 500 times over the corpus, document by document, and word by word. The distance of topics from the global topic, the list of words in the vocabulary, and the corresponding frequency have been computed using the Hellinger and Jaccard distance measurement techniques.

Table 10 shows the average of 100 topics inferred from the corpus before and after lemmatization. The test outcomes found that the distance of topics modeled over the lemmatized corpus is more than that of the unlemmatized corpus. This is because interpretability and semantic coherence are the parameters correlated with a distance of topics from the predefined global topic. Therefore, it can be concluded that topics learned through the lemmatized text are more significant than topics learned through unlemmatized text.

The distance of the topic model before performing the lemmatization of words was compared with lemmatized corpus topic model. Table 10 comprises the experimental outcomes. The distance of lemmatized topics was increased; specifically, the Hellinger distance inscreased by 3% to 5% and the Jaccard distance by more than 2%. It can be inferred that modeling topics in lemmatized text made them more interpretable and meaningful. Moreover, the distance got wider with the shrinkage of the vocabulary size.

**Figure 6:**
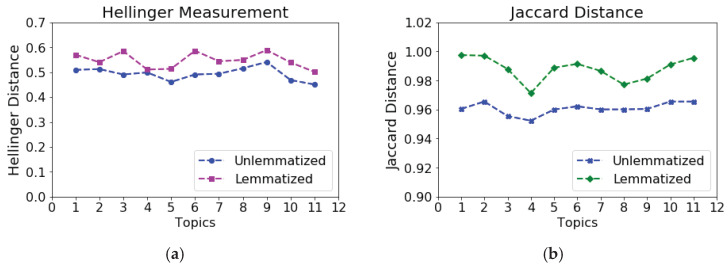
Distance comparison between unlemmatized and lemmatized topics for first 10 topics. (**a**) Hellinger distance measurement; (**b**) Jaccard distance measurement.

Comparing individual distance values, Figure 6a,b depicts the Hellinger and Jaccard distance of 10 topics from the very general topic, respectively. Each topic of lemmatized text falls farther from the global topic than the topics discovered from the unlemmatized text in both cases. The Hellinger distance increased from 1% to 9%, while the Jaccard distance showed a distance difference within the range of 1% to 3%.

### 6.2. The Semantic Coherence Score

We used the Pointwise Mutual Information (PMI), Normalized PMI (NPMI), and Log Conditional Probability methods for the semantic coherence measurement. The semantic coherence of the topics showed improvement after the lemmatization process. All three methods found an increase in the coherence score. In addition to distance measurements, the semantic coherence scores also support that topic models become more interpretable if topics are modeled in the lemmatized text. The coherence value increases as well with the reduction in the size of the vocabulary. The semantic coherence was enhanced up to 3% with LCP and PMI methods and up to 6% for NPMI. Although the topic models found a slight improvement in the semantic coherence values with a decrease in the vocabulary size, the inference time decreased remarkably. Hence, topics learned from lemmatized text are more meaningful than those from unlemmatized text.

**Figure 7:**
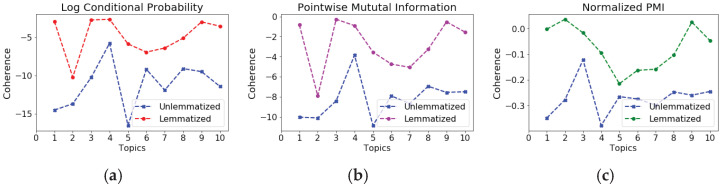
Semantic coherence value comparison between unlemmatized and lemmatized topics for the first 10 topics. (**a**) Log Conditional Probabilities; (**b**) Pointwise Mutual Information; (**c**) Normalized PMI.

We computed the semantic coherence value for 10 topics individually for each technique described. Figure 7a–c depicts the comparison of the coherence value of the unlemmatized text topic model with the lemmatized text topic model. The coherence improvement increased within the range of 2% to 11% for LCP, 2% to 9% for PMI, and up to 1% to 3% for NPMI, while the overall enhancement is lower compared to the 10 topics. However, several topics did not improve semantically. Moreover, the coherence values decreased for a few topics as well. These points caused the average enhancement to have lower values than the first 10 topics.

## 7. Conclusions

In this paper, we have proposed a DFA-based lemmatization approach for Gujarati text. The proposed method comprises more than 59 rules for lemmatizing Gujarati text. We have managed to lemmatize 83% of words correctly. To examine the effect of the proposed lemmatization approach on text analysis, we applied LDA for inferring topics from a text corpus. We showed that the vocabulary size was reduced drastically when lemmatization was involved, although the number of tokens did not decrease. The experimental outcomes revealed that the interpretability of topics increased when the corpus was lemmatized. The topics became more precise and meaningful. This finding was supported by the Hellinger distance and Jaccard distance. Moreover, the semantic coherence measurements supported improving the quality of topics. Our three techniques, PMI, NPMI, and LCP, reported an increase in the coherence value. Furthermore, topics were found to be more specialized when they were modeled from the lemmatized corpus. Moreover, the semantic association among the topics’ words has also been enhanced. A generalized approach can be developed for any text corpus in the future. For example, a set of rules can achieve lemmatization for news articles, discussion forums, textbooks, novels, social media text domains, etc.

## Figures and Tables

**Table 1:** Topics with top 10 words.

Topic 1		Topic 2		Topic 3		Topic 4	
Word	Prob.	Word	Prob.	Word	Prob.	Word	Prob.
Ballot	0.073	NYSE	0.104	Gym	0.064	Fire	0.081
Voting	0.071	Predict	0.082	Guideline	0.062	Fundamental	0.079
Poll	0.069	Profitability	0.082	Diet	0.060	Force	0.077
Booth	0.064	NASDAQ	0.073	Fitness	0.060	Galaxy	0.077
Campaign	0.062	Negotiable	0.073	Grains	0.059	Earth	0.077
Election	0.060	Profit	0.073	Growth	0.059	Experimental	0.075
Democracy	0.057	Peak	0.068	Doctor	0.057	Energy	0.069
Leadership	0.053	Portfolio	0.062	Yoga	0.055	Explosion	0.063
Elector	0.050	Price	0.061	Health	0.055	Star	0.063

**Table 2:** Lemmatization for different languages.

Author	Language	Application	Pub. Year	Approach	Accuracy	No. of Tokens
[59]	Assamese	Word Sense Disambiguation	2022	Rule-based	82	50,000
[60]	Arabic	Annotation	2018	Dictionary-based	98.6	46,018
[44]	Bengali	Word Sense Disambiguation	2016	Rule-based	96.99	6341
[43]	Hindi	Time Complexity	2013	Rule-based	89.02	2500
[49]	French	Pos Tagging	2010	Rule-based	99.28	350,931
[55]	Kazakh	Information Retrieval	2019	Rule-based	N/A	N/A
[46]	Arabic	Lexem Models	2018	Feature Ranking	N/A	N/A

**Table 3:** Stemming and lemmatization.

Word	Stemming	Lemmatization
Information	Inform	Information
Informative	Inform	Informative
Computers	Comput	Computer
Feet	Feet	Foot

**Table 4:** Part 1: Rules for the first word of the sentence.

Sr. No	Rule Name	How Many Letters / Characters to Check	Letters	What to Delete from Word	What to Add after Deletion	Example
1	Check if the last letter is ’ ો ’	last 3 characters	’ ો ’, ’ન’, ’ ો ’,	last 3 characters	NA	મહાપુરુષોનો = મહાપુરુષ
2	Check if the last letter is ’ો ’	last 4 characters	ો ’, ’ન’, ’ઓ’, ’ા’	last 4 characters	’ ો ’	છોકરાઓનો = છોકરો
3	Check if the last letter is ’ો	last 4 characters	’ ો ’, ’ન’, ’ઓ’ not ’ા’	last 3 characters	NA	છોકરીઓનો = છોકરી
4	Check if the last letter is ’ો	last 2 characters	’ ો ’, ’ન’	’ ો ’ and check the remaining word with the words in the n-ending words file. If a match occurs, then print the word; else, remove ’ ો ’, ’ન’	NA	વાહનો = વાહન
5	Check if the last letter is ’ો	last 2 characters	’ ો ’, ’ન’	last 2 characters	NA	સીતાનો = સીતા
6	Check if the last letter is ’ી’	last 3 characters	’ી’, ’ન’, ’ ો	last 3 characters	NA	મહાપુરુષોની = મહાપુરુષ
7	Check if the last letter is ’ી’	last 4 characters	’ી’, ’ન’, ’ઓ’, ’ા’	last 4 characters	‘ ો ’	છોકરાઓની = છોકરો
8	Check if the last letter is ’ી’	last 4 characters	’ી’, ’ન’, ’ઓ’ and not ’ા’	last 3 characters	NA	છોકરીઓની = છોકરી
9	Check if the last letter is ’ી’	last 2 characters	‘ી’, ’ન’	last 2 characters	NA	સીતાની = સીતા
10	Check if the last letter is ’ી’	last 3 characters	’ી’, ’ થ ’, ’ ો ’	last 3 characters	NA	મહાપુરુષોથી = મહાપુરુષ

**Table 5:** Part 2: Rules for the rest of the words of the sentence.

Sr. No	Rule Name	How Many Letters / Characters to Check	Letters	What to Delete from Word	What to Add after Deletion	Example
1	Check if the last letter is ’ુ’:	last 3 characters	‘ુ ’,‘ ય’, ‘્’	last 3 characters	‘વ’, ‘ુ ’	બન્યુ =બનવુ
2	Check if the last letter is ’ુ’:	last 4 characters	’લ’, ’ે’, ’ય’, ’ા’	last 4 characters	‘વ’, ‘ુ ’	સંતાડાયેલુ =સંતાડવુ
3	Check if the last letter is ’ુ’:	last 2 characters	‘ુ ’, ‘વ’	last 2 characters	NA	રમવુ= રમ
4	Check if the last letter is ’ુ’:	last 2 characters	‘ુ ’, ‘ત’	last 2 characters	NA	રમતુ= રમ
5	Check if the last letter is ’ુ’:	last 2 characters	‘ુ ’, ‘મ’	last 2 characters	NA	પાંચમુ = પાંચ
6	Check if the last letter is ’ુ’:	last 2 characters	‘ુ ’, ‘શ’	last 3 characters	NA	આવીશુ = આવ
7	Check if the last letter is ’ુ’:	last 3 characters	’ુ’, ’ન’, ’ ો ’	last 3 characters	NA	મહાપુરુષોનુ = મહાપુરુષ
8	Check if the last letter is ’ુ’ :	last 4 characters	’ુ’, ’ન’, ’ઓ’, ’ા’	last 4 characters	‘ ો’	છોકરાઓનુ = છોકરો
9	Check if the last letter is ’ુ’:	last 4 characters	’ુ’, ’ન’, ’ઓ’ not ’ા’	last 3 characters	NA	છોકરીઓનુ = છોકરી
10	Check if the last letter is ’ુ’:	last 2 characters	’ુ’, ’ન’	last 2 characters	NA	સીતાનુ = સીતા

**Table 6:** Tokens, vocabulary, and TTR.

Preprocessing Steps	No. of Tokens	Vocabulary Size	% of Tokens in Vocabulary	TTR
After tokenization	1,167,630	89,696	7.681885529	0.077
After stopwords removal	870,521	89,003	10.22410717	0.102
After punctuation removal	746,292	889.87	11.92388502	0.119
Alphanumeric to alphabetic word	746,292	86,271	11.5599524	0.116
After single-letter word removal	620,133	86,098	13.8837959	0.139
After lemmatization	620,133	50,043	8.069720528	0.081

**Table 7:** Single letter words.

Word	Probability	Word	Probability
’કે’ *(Kē / Whether)*	0.003833333	’જો’ *(Jō / If)*	0.000750000
’છે’ *(Chhē / Is)*	0.025166667	’જ’ *(Ja / Only)*	0.002500000
’જે’ *(Jē / Whom)*	0.001083333	’ન’ *(Na / No)*	0.001000000
’તે’ *(Tē / That)*	0.001666667	’બે’ *(Bē / Two)*	0.001166667
’એ’ *(Ē / That)*	0.000833333	’તો’ *(Tō / Then)*	0.001833333
’આ’ *(Ā / This)*	0.004250000	’હું’ *(Huṁ / I)*	0.000416667
’છો’ *(Chho / Is)*	0.000833333	’શ્રી’ *(Shree / Mr.)*	0.001583333

**Table 8:** Global topic or overly general topic.

Word	Frequency	Word	Frequency
ટેક્સ (*Ṭēksa*/Tax)	231	જાહેર (*Jāhēra*/Public)	67
વરસાદ (*Varasāda*/Rain)	191	પ્રોજેક્ટ (*Prōjēkṭa*/Project)	57
ગુજરાત (*Gujarāta*/Gujarat)	189	રકમ (*Rakama*/Amount)	46
જાહેર (*Jāhēra*/Public)	182	જમીન (*Jamin*/Soil)	45
સરકાર (*Sarakāra*/Government)	170	યોગ (*Yoga*/Yoga)	39
યોગ (*Yōga*/Yoga)	147	ગુજરાતમાં (*Gujarātamām*/In Gujarat)	35
શરૂ (*Śarū*/Start)	138	પ્લાન (*Plan*/Plan)	31
ભારતીય (*Bhāratīya*/Indian)	136	વર્ષે (*Varṣē*/Year)	30
ભારત (*Bhārata*/India)	126	શક્તિ (*Śakti*/Power)	27
બુલેટ (*Bulēṭa*/Bullet)	121	એફઆઇઆઈ (*Ēpha’ā’ī’ā’ī*/FII)	25
પાણી (*Pāṇī*/Water)	118	સમયસર (*Samaysara*/On time)	25
અમદાવાદ (*Amadāvāda*/Ahmedabad)	114	મહત્ત્વ (*Mahtava*/Importance)	24
પ્રવેશ (*Pravēśa*/Entry)	112	વિધુર (*Vidhura*/Widower)	19
તલાક (*Talāka*/Divorce)	112	મુંબઈ (*Mumba*/Mumbai)	18
સ્માર્ટફોન (*Smārṭaphōna*/Smartphone)	108	⋯	⋯
નિર્ણય (*Nirṇaya*/Decision)	107	⋯	⋯
બાહુબલી (*Bāhubalī*/Bahubali)	106	⋯	⋯

**Table 9:** Interpretable topica.

Word	Frequency	Word	Frequency
ધર્મ (*Dharma*/Religion)	86	સાક્ષાત (*Sākṣāta*/Confirmed)	18
આનંદ (*Ānanda*/Happiness)	41	પૂજાપાઠ (*Pūjāpāṭha*/Worship)	18
ઈશ્વર (*Īśvara*/God)	37	જાગૃતિ (*Jāgrti*/Awareness)	18
યહુદી (*Yahudī*/Jew)	32	સાંપ્રદાયિક (*Sāmpradāyika*/Sectarian)	18
કર્મકાંડ (*Karmakāṇḍa*/Ritual)	22	પ્રેમ (*Prēma*/Love)	18
નૈતિક (*Naitika*/Moral)	21	ખ્રિસ્તી (*Khristī*/Christian)	16
શ્રધ્ધા (*Śrad’dhā*/Devotion)	21	ઇસ્લામ (*Islāma*/Islam)	16
આધ્યાત્મિક (*Ādhyātmika*/Spiritual)	20	જીવન (*Jīvana*/Life)	9
ધાર્મિક (*Dhārmika*/Religious)	20	પ્રત્યે (*Pratyē*/Towards)	8
મુલ્યો (*Mulyō*/Values)	19	માણસ (*Māṇasa*/Human)	8

**Table 10:** The distance between topics for the unlemmatized and lemmatized corpus.

No. of Tokens	Vocabulary	Inference Time (in Seconds)	Distance Measurement			
			Unlemmatized		Lemmatized	
			Hellinger	Jaccard	Hellinger	Jaccard
604,389	85,463	33.14	0.476	0.970	0.491	0.993
561,648	42,722	29.63	0.495	0.968	0.546	0.998
531,870	27,533	26.77	0.481	0.982	0.520	0.996
512,085	21,238	22.15	0.489	0.982	0.517	0.999
496,373	17,310	18.92	0.495	0.982	0.528	1.000
483,108	14,657	16.55	0.492	0.983	0.526	0.999

## Data Availability

No data available to carry out this research.
